# Vulnerability and Adaptation to Extreme Heat in Odisha, India: A Community Based Comparative Study

**DOI:** 10.3390/ijerph16245065

**Published:** 2019-12-12

**Authors:** Subhashisa Swain, Shreeporna Bhattacharya, Ambarish Dutta, Sanghamitra Pati, Lipika Nanda

**Affiliations:** 1 School of Medicine, University of Nottingham, Nottingham NG5 1PB, UK; 2 Indian Institute of Public Health, Bhubaneswar, Public Health Foundation of India 267/3408, Mayfair Road, Jayadev Vihar, Bhubaneswar, Odisha 751013, India; 3 Regional Medical Research Center, Indian Council of Medical Research, Bhubaneswar, Odisha 751017, India

**Keywords:** heat, vulnerability, India, coping mechanism

## Abstract

Background: Extreme heat and heat illness are becoming very frequent in India. We aimed to identify the factors associated with heat illness and the coping practices among city dwellers of Odisha, India during the summer. Methods: A cross-sectional study included 766 households (HHs) in twin cities of Odisha covering a population of 1099 (slum: 404 and non-slum: 695) in the year 2017. We collected information on sociodemographic, household characteristics, coping practices to heat and the heat illness history reported during the summer. Multivariate logistic regression accounting for clustering effects at the household and slum levels was used to identify the associated factors of heat illness after adjustment of other variables. Result: Nearly, 49% of the study participants were female and the mean age was 38.36 years (95% confidence interval (CI): 37.33–39.39 years). A significant difference of living environment was seen across the groups. More than two-thirds of the study participants at least once had heat illness. In the non-slum population, males (adjusted odds ratio (aOR): 3.56; 95% CI: 2.39–5.29), persons under medication (aOR: 3.09; 95% CI: 1.15–8.29), and chronic conditions had higher association with heat illness. Whereas, in the slum population, having a kitchen outside the home (aOR: 1.63; 95% CI: 1.02–3.96) and persons with chronic conditions were positively associated with heat illness. Use of cooling practices in slum areas reduced the risk of heat illness by 60%. Conclusion: Heat illness is associated with the living environment and physical health of the individuals. Identifying the vulnerable population and scaling up adaptive practices can strengthen the public health preparedness.

## 1. Introduction

A heat wave (HW) leads to physiological stress, which sometimes can claim a human life [1]. Physiologically, whenever the environmental temperature increases above 37 °C along with high humidity the human body starts gaining heat from the atmosphere leading to heat illness [2]. In India the definition of HW varies across the geographical areas with different possible ranges of temperature [3]. It is evident that, not only the temperature, but also humidity, wind, and other climatic factors can play a greater role in defining the extreme heat condition [4]. Extreme heat during summer is an emerging threat to biodiversity, economy, livelihood, and the future generation. This change in environmental conditions is becoming a norm rather than an exception throughout the world [5,6].

In July 1995, a HW killed more than 700 people in Chicago [7], and in August of 2003, it took more than 70,000 lives in Europe [8]. Extreme heat has been found to be the deadliest weather-related hazard in some locations. Every year, because of the heat, India loses thousands of people during the summer. For example, in a Western city of India, the heat-related death count in the year 2010 was nearly 1400 [9] and in 1998, Odisha, an Eastern state of the country, lost 2042 people [10]. It is predicted that the average temperature across the globe during the summer is going to increase over the years, thus in the future, extreme heat days are unavoidable [11].

Responsiveness to extreme heat depends on the existing national and regional policies and existing coping mechanism. Heat-related illness is dependent on individual and community-level attributes such as, sociodemographic, income, medical conditions, occupation, behavioral habits, neighborhood, and community practices. Thus, an important task in preventing death and morbidity are to identify the ‘at-risk’ or vulnerable population. Researchers have developed a ‘vulnerability index’ to identify the ‘at-risk’ population [12]. However, the majority used climatic factors [13]. Nayak et al. found 13 social vulnerability factors for New York state which includes ethnicity, income, place of living, ageing, and disability [12]. Inclusion of sociodemographic, environmental, and other individual health factors in such an index can help specific public health preparedness [14].

Available evidence on heat exposure in India is not comprehensive and is mostly confined to heat-related illness at workplace [15,16]. Ageing, chronic conditions, and occupation have been reported to be associated with heat morbidity [17]; but geographical, demographic, and climatic diversities within the country demand further exploration of vulnerable factors such as living conditions in community settings. Even though Odisha has had a policy on extreme heat since 2000 [18], the morbidity of heat illness is still high. To date, no scientific study has been done to identify the ‘at-risk’ population and to understand the adaptation mechanism in practice in the context of living environment and income. We aimed to identify the factors associated with heat illness and their differences across slum and non-slum areas of two cities of Odisha, India.

## 2. Methods

This study was done in two cities of the state in 2017 from June to August.

Study settings: Bhubaneswar and Cuttack are two of the largest cities (twin cities) of Odisha, situated nearly 60 km away from the Bay of Bengal in Eastern India [19]. In 2011, the population of the twin cities was nearly 13 million, of which 301,611 were residing in slum areas [20,21]. Both cities have a summer period from April to June of each year followed by four months of rainy season. In summer, the maximum and minimum temperature averaged around 37 °C and 26 °C, respectively [22]. The cities have meteorological stations collecting information on temperature, humidity, rainfall, and other climatic factors. There is no wide variation of these parameters found between and within the cities. However, the population density and housing structures and other social determinants are not uniformly distributed. The association of climatic factors with mortality in these cities has been described elsewhere [23].

Sample size: Even though our primary objective was not to estimate the prevalence of heat illness, in the absence of appropriate literature on risk factors for heat illness, we used the prevalence value for sample size estimation. According to the literature, nearly 46.3% of the sample households (HHs) had heat-related illness [17]. Expecting a similar proportion in our study, with 5% error and 80% power and a design effect of 1.8 (because of clustering design), the required HHs for our study were 688. Considering the non-response rate of 10%, final estimated HHs were 764. An equal number of HHs were recruited in each city (per city, HHs = 382).

Sampling: Stratified clustering sampling method was used for the selection of wards. Firstly, we listed the wards of the twin cities (total = 126; Bhubaneswar = 67 and Cuttack = 59) and randomly selected 13 wards from each city (total 26 wards, 20% coverage). Secondly, from each selected ward, one slum area and adjacent non-slum area were randomly selected. The adjacent non-slum area was chosen for better comparison adjusting for the geographical and climatic variation. Thirdly, within each selected area 29 HHs were chosen through systematic random sampling method. The first house was selected randomly, then every fourth house on right side of the interviewed HHs was included until we covered the locality. According to available data, the average number of HHs in one locality varied from 80–100. For over 382 HHs in each city we covered nearly 15 HHs per area (= 382/26). Details of the sampling design are given in Supplementary Figure S1.

From each HH, we included all individuals who spend most of the time residing inside their home during the summer. For example, women who do not perform outdoor work, pregnant/lactating women, elderly people (≥60 year), children (<5 year), persons with disability (physical or mental), students (because they stay at home during summer holidays in school), persons with chronic diseases, any member suffering from immunocompromised diseases, and/or persons with debilitating conditions. The list of possible vulnerable populations was developed by the research team after extensive literature research.

Data collection tools: Two separate data collection tools (one each for HH and individual) were used in the vernacular language Odia (translated and back translated to English). It was piloted among an eligible non-study population of 30. The individual level questions were on demographics, health conditions (pre-existing and heat-related symptoms and diagnoses), and occupational settings. Household questions collected information regarding behaviors and practices. The summary of the questionnaire is given below, and the full version can be obtained with request from the authors.

Sociodemographic: In this section, most of the questions were close-ended. For each vulnerable person in the HH, information was collected on age, sex, highest level of education, present major occupation (involved during summer), self-reported preexisting health conditions, medication history (chronic medication), and any heat-related illnesses and symptoms.

Household: Household questions elicited information on type of house, type of roofing, electricity at home, location of kitchen and ventilation status (checked by the researchers), any ‘electricity cut’ during the summer, source of water supply for general purposes and drinking, source of cooking fuels, and cooling mechanisms in practice. The household adaptation mechanism to heat during summer was explored through an open question “At home, what are the methods you adopt to get protection from heat during summer?”

Heat-related information: The participants were asked about the harmful effects of heat, their last experience of extreme heat, source of information on heat forecasting, source of information on ‘how to protect from heat?’, visits to health facilities because of heat-related illness, mode of visit, and suggestions to improve the extreme heat condition.

The main outcome of the study was self-reported heat-related symptoms (HRS) and physician diagnosed heat-related illnesses (HRI) experienced in the past six months. Respondents were asked if they had any of the listed episodes: ‘heat cramp’, ‘heat exhaustion’, ‘heat syncope’, ‘heat rash’, and ‘heat stroke’. Detailed definitions used for each of these are given in the supplementary file. We combined all the responses to create a single binary variable ‘heat illness’ with “yes” defining anyone experiencing any of those symptoms or illnesses.

The survey was carried out by four trained researchers with expertise in community surveys. We preferred to interview the female head of household to report the presence of any vulnerable family members, the housing conditions and coping practices. For children/students and elderly people, a female representative of the house responded to the questions (mostly by mother/young women). Average time taken for each survey was 45 minutes. After completion of the survey each household was provided with an information sheet on do’s and do not’s during summer in the vernacular language.

Primary outcomes of interest included self-reported heat-related symptoms or illness. To increase analysis power, symptom and illness options were condensed into a single binary variable, where “yes” corresponded to ever experiencing any of those symptoms or illnesses. The duration of heat-related illness and healthcare utilization during last episode was inquired.

Ethical statement: Both written and verbal consent was obtained from the study participants before the interview. For minors aged less than 18 years, parental consent was obtained.

Statistical analysis: All analyses were performed using STATA SE 12 (Stata Corp., Texas, USA). For descriptive purposes, variables were categorized as demographics, exposure variables, susceptible factors, adaptive behaviors, and outcomes. Chi-squared test was done to test the significant association between the HHs and individual characteristics with the area of living (slum and non-slum). A comprehensive list of practices of rescue during summer were tabulated across the group and tested for statistical significance. Based on the signs and symptoms experienced, the respondents were classified into different heat-related illnesses. As a result of the survey design of data, we used ‘svy’ command for our analysis. Univariate regression was done with each variable, those found to be significant (*p* < 0.05) were included in the final model. Multivariate logistic regression using generalized estimating equations (GEE) to account for clustering effects at the household and slum levels was performed to test the hypothesis for overall study population and stratified analysis for slum and non-slum areas.

## 3. Results

We visited 800 HHs and information was collected from 766 HHs (response rate was 95.75%) and 1099 vulnerable individuals. Total HHs included 306 slum (80% response rate) and 460 non-slum HHs with 404 and 695 individuals from each area, respectively. The unproportioned distribution was due to a greater non-response rate in slum areas compared to non-slum, so we decided to achieve the estimated sample size by recruiting more HHs from non-slum areas. The reasons of non-response in slum areas were absence of HH leaders (after three visit attempts, *n* = 26), lack of willingness to participate (*n* = 12), incomplete information provided (*n* = 17), and non-vulnerable people residing in the house (*n* = 31).

Nearly 49% participants were female and more than half of them were from slum areas. The mean age of study participants was 38.36 years (95% confidence interval (CI): 37.33–39.39 years). Table 1 describes the detailed sociodemographics. Nearly 50% of houses in slum areas had a tin/asbestos roof compared to 3% in non-slum areas, which was statistically significant at *p*-value < 0.05. Two-thirds of slum houses had an electricity supply and of them, nearly 65% experienced a power cut during the summer. More than 84% of slum HHs had access to regular water supply during summer. All the household characteristic differences were statistically significant (Table 1).

**Figure 1:**
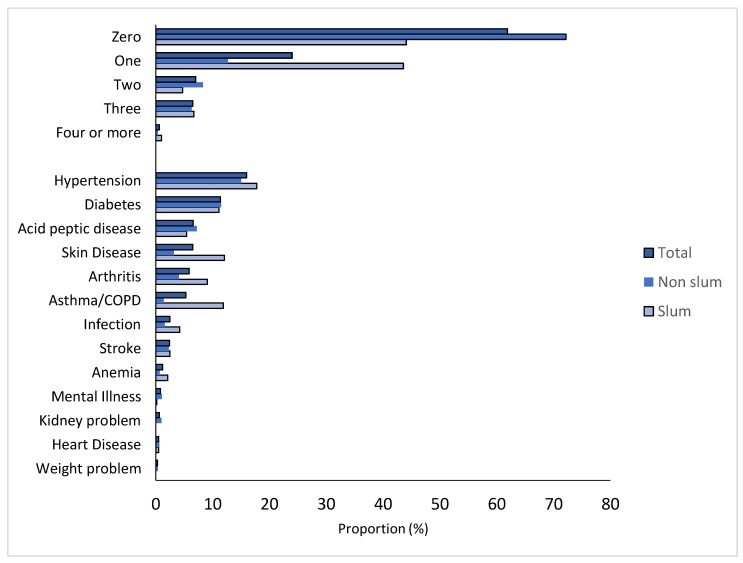
Distribution of morbidity count and pattern across the groups (total = 1099; slum = 404; non-slum = 695). COPD: chronic obstructive pulmonary disease.

**Figure 2:**
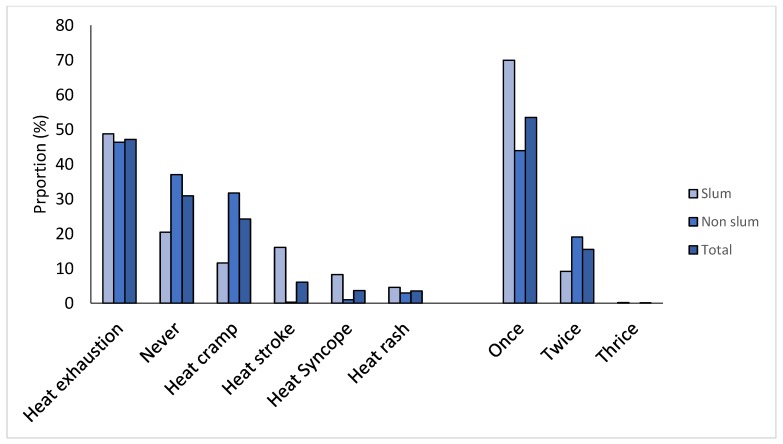
Heat-related illness types and count reported in different groups (total = 1099; slum = 404; non-slum = 695).

Two-fifths of the non-slum and two-thirds of the slum participants had at least one chronic condition. No statistical difference was seen for the prevalence of hypertension, diabetes, acid peptic diseases, and stroke. The proportion of people with two or more chronic conditions was higher in the non-slum population (Figure 1). More details of conditions are given in Table S1. Nearly 25% of the people in both groups reported to be on medication for any of their chronic illnesses. Table 2 describes the adaptive/coping mechanism in both groups. Drinking water, wearing wet clothes, covering the roof, and wearing light/cotton clothes were leading practices. No statistical significance was seen for bathing multiple times, wearing wet clothes, eating rice water, and drinking water (Table 2). Heat exhaustion, heat cramps, and heat stroke were the leading heat-related events reported by participants (Figure 2). Details of the heat-related illness across groups are given in Table S2.

Multivariate logistic regression did not show significant association between place of residence and heat illness. Males staying indoors were at two times higher risk of getting heat illness compared to females. Presence of a kitchen outside the home made the residents 25% more vulnerable towards heat exposure and illness. Chronic conditions predisposed higher risk (2–4 times) of getting heat illness. Practice of cooling methods like use of fan/air conditioner/cooler decreased the chance of getting heat illness by 60%. Among people from the slum area, the presence of a kitchen outside the home, and presence of chronic conditions were associated with heat illness.; whereas, among non-slum people, males, presence of chronic conditions, and people currently under medication had higher association with heat illness (Table 3).

## 4. Discussion

To our knowledge, this is the first ever study reporting the adaptive practices during extreme heat and associated factors of heat illness in Eastern India. Key findings from our multivariate analysis are as follows: (1) males staying indoor were two times more likely to get heat illness compared to females; (2) presence of a kitchen outside the home was two times more likely to be association with heat exposure and illness; (3) presence of chronic conditions predisposes higher risk (2–4 times) of getting heat illness; and (4) practice of cooling methods like the use of fan/air conditioner/cooler decreased the chance of getting heat illness by 60%. Participants from slums were more dependent on water and other traditional cooling mechanisms, whereas, in non-slum areas, personal protection and architectural modifications were common in practice.

Housing conditions play an important role in heat trapping, such as inadequate ventilation, tin roof, and using solid fuel for cooking and add to the temperature during the summer. Along with that, crowding and dense populations concentrated at smaller areas increase the housing temperature. However, we did not find significant association with place of residence. It is contradictory with findings from other studies [17,24,25]; so, we assume it is not simply the place of residence, but individual and housing factors that contribute to heat illness. Previous studies have focused only on slum areas without a comparison population. Our study is the first ever to present a comparative statistic.

Commonly, males are more exposed to heat because of their nature of occupation and outdoor activities [24]. Increased duration of exposure to heat at work has been supported by other studies [16]. In our study, males in non-slums were mostly elderly people or students who had a higher chance of having heat illness. Perhaps, elderly men have chronic conditions and functional impairment which makes them vulnerable towards heat. However, this combined association of men and chronic conditions needs further investigation.

Another significant finding from our study was the presence of kitchens outside the home, which increases the atmospheric temperature and becomes a threat. In India, usually people cook during the day, so having the kitchen outside contributes to the problem [26]. This association was more prominent among slum people, justifying the explanation. People living in slum areas use different solid fuels for cooking in the daytime. The temperature and gas generated by these fuels add to the ambient temperature, increasing the risk of heat illness.

The presence of chronic conditions makes a person frail and decreases the tolerance capacity towards heat. In general, people with chronic conditions are under regular medication. Some of these medicines increase physiological temperature, such as antipsychotic drugs [27]. Management of chronic conditions, such as chronic kidney disease, follows a regulated diet and hydration. Patients on such medications, when exposed to heat, become easily dehydrated. So, not only merely the disease alone, but also multiple factors increase the complexities and risk of heat illness. The relationship between chronic conditions, medications, and heat illness was previously studied [13]. The association of chronic conditions with heat illness was significant in both groups. Thus, studying individual factors with physiological factors in identifying vulnerable people towards heat is necessary.

The differences in cooling practices in both groups were significant. People using any cooling methods had nearly 60% less of a chance of having heat illness. In slum areas, people used methods such as spreading straws on roof to protect the houses from heat, whereas, in non-slum areas ‘albedo’ painting of the house was common in practice. Practices in slum areas are mostly associated with use of water in various ways like drinking and spraying. These adaptive practices are directly linked with income, availability of water, and cultural practices. In Odisha, people from rural areas, during summer eat ‘rice water’ to keep themselves cool. However, people in non-slum areas prefer architectural modification or cotton clothing to get the respite from the heat. Studies from other cities have shown similar findings [17]. These variations in adaptive mechanisms need to be studied in detail for designing appropriate intervention strategies and expanding the heat action plan to other areas.

Being a cross-sectional survey-based study, some inherited biases cannot be ignored such as, reporting and recall bias, interviewer bias, and biases in the survey instrument. As we asked respondents about health conditions of other members, illness history, and medication practices, some extent of under reporting cannot be ignored. Lack of information on exposures can be another limitation, for which we could not establish proper causal association. Measurement of heat illness was purely based on the signs and symptoms told by the participants, so misclassification biases cannot be ruled out. Similarly, self-reported illness could have underestimated the actual burden considering the underdiagnoses of diseases especially in developing countries. We used the sampling design effect during our analysis to adjust for cluster sampling. We did not collect spatio-temporal information regarding heat illness. However, as the households were selected from same area, the natural adjustment of climatic factors was assumed. We did not have detailed information on outside home kitchens in relation to heat illness. The study was retrospective in nature, so it was difficult to obtain the exact date and temperature of the day heat illness occurred. However, we presented the average temperature and humidity of the cities during the heat illness reported months (Table S3).

## 5. Conclusions

Residents of slum communities are more exposed to extreme heat, more susceptible to heat illness, and have fewer adaptation options available. With predicted increase in the frequency and intensity of extreme heats, targeted policy interventions coordinated across multiple levels are needed to reduce the devastating health effects of heat stress. An awareness program must consider the chronic conditions and develop a tailor-made guidance. Coping strategies helpful in reducing the heat illness should be encouraged to practice and can be adapted for other vulnerable areas too. Further studies must be done to understand the fabrics of heat illness with sociocultural practices with respect to spatiotemporal variations.

## Figures and Tables

**Table 1:** Sociodemographic and housing characteristics.

**Variable**	**Category**	**Slum (*n* = 404) Percentage (95% CI)**	**Non Slum (*n* = 695) Percentage (95% CI)**	**Total (*n* = 1099) Percentage (95% CI)**	***p*-Value ^#^**
Sex	Female	53.2 (49.8–57.2)	46.5 (42.8–50.2)	49.0 (46.0–51.91)	0.031 *
Caste	Schedule Caste	59.4 (54.5–64.1)	44.1 (40.4–47.7)	49.7 (46.7–52.6)	<0.001 *
	Schedule Tribe	17.6 (14.1–21.6)	4.3 (3.1–6.1)	9.2 (7.6–11.1)	
Religion	Hinduism	92.1 (89.0–94.3)	98.7 (97.5–99.3)	96.3 (94.9–97.2)	<0.001 *
	Islam	5.9 (4.0–8.7)	0.9 (0.4–1.9)	2.7 (1.9–3.8)	
	Christian	1.5 (07–3.3)	0.4 (0.1–1.3)	0.8 (0.4–1.56)	
Age group (in years)	≤5	11.9 (9.1–15.4)	2.7 (1.7–4.2)	6.1 (4.8–7.7)	<0.001 *
	6–15	5.7 (3.8–8.4)	5.6 (4.1–7.6)	5.6 (4.4–7.2)	
	16–30	18.1 (14.6–22.2)	23.5 (20.3–26.7)	21.5 (19.1–24.0)	
	31–45	30.9 (26.6–35.6)	32.4 (29.0–4.0)	31.8 (29.1–34.7)	
	46–60	19.6 (15.9–23.7)	28.3 (25.1–31.8)	25.1 (22.6–22.8)	
	>60	13.9 (10.8–17.6)	7.5 (5.7–9.7)	9.8 (8.2–11.7)	
**Household Characteristics**		**Slum (*n* = 306) Percentage (95% CI)**	**Non Slum (*n* = 460) Percentage (95% CI)**	**Total (*n* = 766) Percentage (95% CI)**	***p*-Value**
Housing	Kutcha	57.2 (52.3–61.9)	0.4 (0.1–1.3)	21.3 (18.9–23.8)	<0.001 *^,#^
	Semi-Pucca	21.5 (17.8–25.8)	0	7.9 (6.4–9.7)	
	Pucca	21.3 (17.6–25.6)	99.6 (98.7–99.9)	70.8 (68.1–73.4)	
Ventilation adequate	Yes	6.4 (4.4–9.3)	98.3(97.0–99.1)	64.5 (61.6–67.3)	<0.001*
Electric supply	Yes	66.3 (61.6–70.8)	99.6 (99.2–99.9)	87.6 (85.5–89.4)	<0.001 *
Power cut	Yes	28.0 (23.8–32.5)	98.7 (97.5–99.3)	72.7 (70.0–75.2)	<0.001 *
Kitchen inside home	Yes	69.1 (64.4–73.4)	99.1 (98.1–99.6)	88.1 (86.0–89.9)	<0.001 *
Regular water supply in summer	Yes	61.6 (56.8–66.3)	98.3 (97.0–99.1)	84.8 (82.5–86.8)	<0.001 *
Wooden chulha for cooking	Yes	74.1 (69.5–78.1)	10.5 (8.4–13.1)	66.2 (63.2–68.9)	<0.001 *
Liquified petroleum gas (LPG)	Yes	49.1 (44.–53.5)	99.23 (97.0–99.8)	66.3 (62.9–69.6)	<0.001 *
Kerosene stove	Yes	55.3 (50.9–59.6)	6.5 (4.04–10.2)	38.5 (35.1–42.0)	<0.001 *
Drinking water	Borewell	13.3 (10.6–16.6)	0.4 (0.01–2.7)	8.9 (7.1–11.1)	<0.001 *
	Piped water supply	81.9 (78.2–85.0)	96.9 (94.0–98.5)	87.1 (84.5–89.3)	
	Open well	4.8 (3.2–7.1)	2.7 (1.3–5.5)	4.1 (2.8–5.7)	
Roofing	Tin/asbestos	43.3 (39.0–47.7)	3.1 (1.5–5.9)	29.5 (26.4–32.8)	<0.001 ^#^
	Cemented	2.0 (1.1–3.7)	96.9 (94.1–98.5)	34.6 (31.3–38.0)	
	Husk/bamboo	49.5 (45.1–53.9)	0	32.5 (29.3–35.9)	
	Other (polythene)	5.2 (3.5–7.5)	0	3.4 (2.3–4.9)	

* Chi-squared test; *p*-value was significant at the level <0.05; ^#^ Fisher-exact test. CI: confidence interval.

**Table 2:** Adaptive mechanism across the groups.

		Slum (*n* = 404) Percentage (95% CI)	Non-Slum (*n* = 695) Percentage (95% CI)	Total (*n* = 1099) Percentage (95% CI)	*p*-Value
Available mechanism	Fan	59.7 (54.7–64.3)	97.4 (95.9–98.4)	83.5 (81.2–85.6)	<0.001 *^,#^
	Cooler	0	3.3 (2.2–4.9)	2.1 (1.4–3.1)	
	A/C	0	13.1 (10.8–15.8)	8.3 (6.8–10.1)	<0.001 *
Practices	Green space	28.5 (24.2–33.0)	99.1 (98.1–99.6)	73.2 (70.4–75.7)	<0.001 *
	Bathing multiple times	4.9 (3.2–7.5)	3.5 (2.3–5.1)	4.0 (3.0–5.3)	0.222
	Resting in open areas	11.9 (9.1–15.4)	2.9 (1.9–4.4)	6.2 (4.9–7.8)	<0.001 *
	Use clay pot for drinking water	8.2 (5.9–11.3)	3.6 (2.4–5.3)	5.3 (4.1–6.8)	0.001 *
	Wet floor and wall	9.4 (6.9–12.7)	3.5 (2.3–5.1)	5.6 (4.4–7.2)	<0.001 *
	Wear wet clothes	16.1 (12.8–20.0)	16.7 (14.1–19.6)	16.5 (14.4–18.8)	0.795
	Wear cotton clothes	0.5 (0.1–1.9)	52.9 (49.2–50.8)	33.7 (30.9–36.5)	<0.001 *
	Use fan/cooler/AC	2.0 (1.0–3.9)	12.5 (10.2–15.2)	8.6 (7.1–10.4)	<0.001 *
	Stay indoors	11.6 (8.8–15.1)	0.9 (3.9–1.9)	4.8 (3.7–6.3)	<0.001 *
	Drink soft drinks (cold)	0.5 (0.1–1.9)	11.7 (9.5–14.3)	7.6 (6.1–9.3)	<0.001 *
	Spread straw/husk	29.7 (25.4–34.3)	13.4 (11.0–16.2)	19.4 (17.1–21.8)	<0.001 *
	Albedo painting on roof	0	30.2 (26.9–33.7)	19.1 (16.9–21.5)	<0.001 *
	Eat rice water	11.5 (9.3–14.1)	9.4 (6.9–12.7)	10.7 (9.1–12.7)	0.277
	Drink enough water	22.8 (18.9–27.1)	24.7 (21.7–28.1)	24 (21.6–26.6)	0.46
	Rest in shade	8.4 (6.1–11.5)	0.4 (0.1–1.3)	3.4 (2.5–4.6)	<0.001
Count	No methods	11.4 (9.2–13.9)	11.3 (8.6–14.9)	11.4 (9.6–13.4)	<0.001
	One method	36.6 (32.1–41.5)	19.1 (16.4–22.2)	25.6 (23.1–28.2)	
	Two methods	43.1 (38.3–47.9)	39.7 (36.1–43.4)	40.9 (38.1–43.9)	
	Three or more	8.9 (6.3–11.8)	29.8 (26.1–32.8)	22.2 (19.4–24.3)	

^#^ Chi-squared test; * *p*-value was significant at the level <0.05; CI: confidence interval; A/C: air conditioner.

**Table 3:** Multivariate adjusted logistic regression for identifying factors responsible for heat-related illness across the place of residence.

Variables	Categories	Total Adjusted OR (95% CI)	Non-Slum (*n* = 695) Adjusted OR (95% CI)	Slum (*n* = 404) Adjusted OR (95% CI)
Type of area	Non-slum	Reference		
	Slum	2.52 (0.96–6.61)	-	-
Sex	Female	Reference	Reference	-
	Male	2.07 (1.51–2.84) *	3.56 (2.39–5.29) *	
Housing type	Kutcha	Reference	-	-
	Semi Pucca	0.89 (0.37–2.16)		
	Pucca	1.20 (0.55–2.60)		
Kitchen	Inside the house	Reference	-	Reference
	Outside of the house	2.42 (1.17–6.00) *		1.63 (1.02–3.96) *
Electricity supply	No	Reference	-	-
	Yes	1.12 (0.54–2.31)		
Power cut during summer	No	Reference	-	Reference
	Yes	1.38 (0.57–3.17)		2.02 (0.68–5.94)
Count of cooling practices	Nil	Reference	-	Reference
	Only one	0.42 (0.18–0.94) *		0.35 (0.15–0.79) *
	Two or more	0.31 (0.13–0.74) *		0.29 (0.12–0.66) *
Presence of wooden chulha	No	Reference	-	-
	Yes	0.95 (0.59–1.51)		
Chronic disease	Nil	Reference	Reference	Reference
	Only one	2.69 (1.67–4.34) *	2.43 (1.24–4.77) *	2.29 (1.24–4.21) *
	Only two	4.64 (2.08–10.35) *	3.04 (1.29–7.14) *	1.76 (0.92–3.95)
	Three	2.74 (1.41–5.32) *	4.31 (1.80–10.35) *	1.47 (0.63–3.42)
	Four or more	4.02 (0.49–32.78)		1.53 (0.35–6.76)
Medication use currently	No	Reference	Reference	-
	Yes	1.29 (0.72–2.33)	3.09 (1.15–8.29) *	

* *p*-value was significant at the level <0.05; CI: confidence interval; OR: odds ratio. Variables significant in univariate logistic regression in each group are included in final model.

## Supplementary Materials

The following are available online at https://www.mdpi.com/1660-4601/16/24/5065/s1, Figure S1: Sampling strategy of the study, Table S1: List of chronic conditions reported across the group, Table S2: List of heat illness across the group, Table S3: Climatic data of study cities during the past four months (summer) of the study period.

## Abbreviations

aOR	adjusted odds ratio
CI	confidence interval
GEE	generalized estimating equation
HH	household
HRI	heat-related illness
HRS	heat-related syndrome
HW	heat wave.
