# Supplementary material for: Vulnerability and Adaptation to Extreme Heat in Odisha, India: A Community Based Comparative Study
Source: Int J Environ Res Public Health. 2019 Dec 12;16(24):5065. doi: 10.3390/ijerph16245065 (PMC6950623; doi:10.3390/ijerph16245065)
Supplement: Supplementary file 1 [file ijerph-16-05065-s001.pdf]

Supplementary file

Definition of 'heat illness'

Heat-related symptoms and illnesses: small blisters or pimples, dry mouth, fatigue, leg cramps, heavy sweating, intense thirst, rapid heartbeat, headache, and leg swelling.

Heat cramp: any painful involuntary brief muscle cramps, spasm or jerk due to heat

Heat exhaustion: fatigue and collapse resulting from prolonged exposure to excessive or unaccustomed heat

Heat Syncope: fainting or fell-down or go senseless because of excessive heat

Heat Rash: an irritation of the skin that results from excessive sweating during hot and humid weather. It can be small blisters or pimples

Heat Stroke: fever and often by unconsciousness when exposed to excessively high temperatures.

Supplementary Figure S1. Sampling strategy of the study.

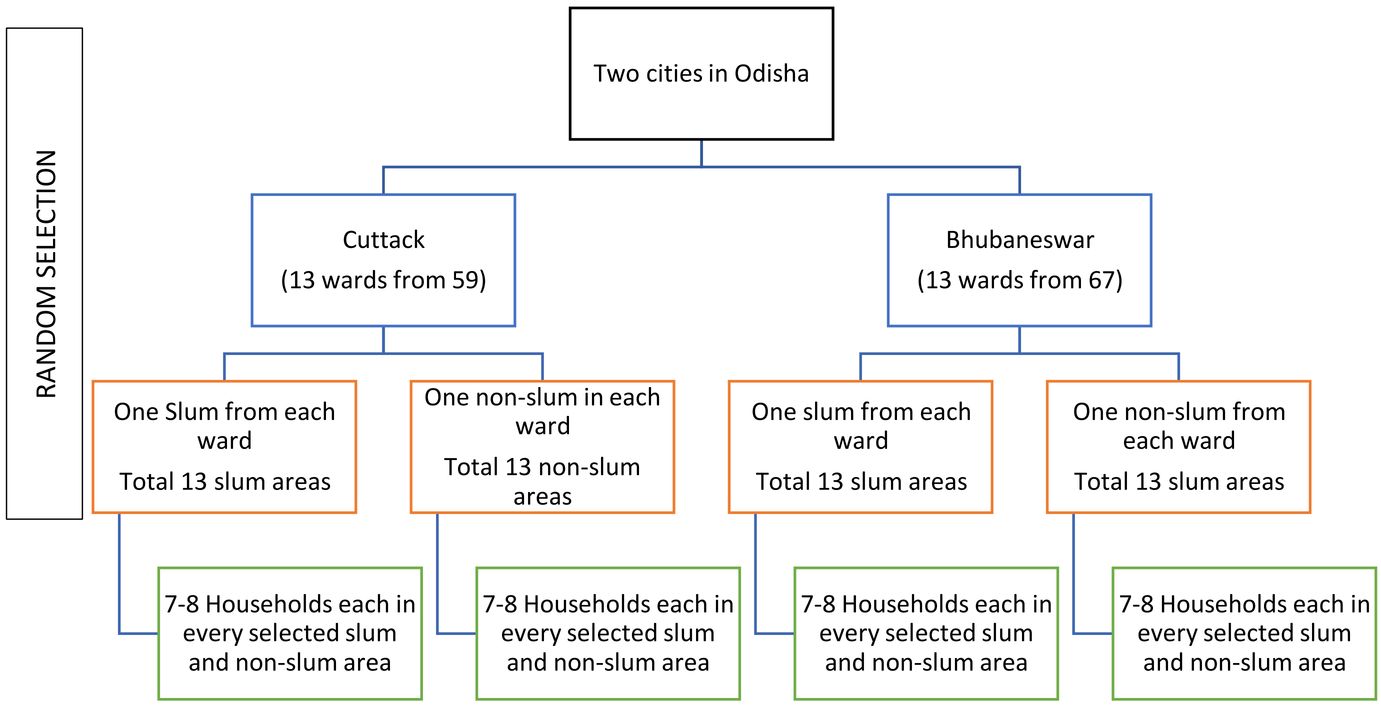

Firstly, we listed the wards of twin city (total =126; Bhubaneswar=67 and Cuttack=59) and randomly selected 13 wards from each city (total 26 wards) covering 20% of total wards. Secondly, from each selected ward, one slum area and adjacent non-slum area was again randomly selected. We chose an adjustment non-slum area for better comparison and adjusting for geographical variation. Thirdly, within each selected area 29 HHs were chosen through systematic random sampling method. First house was selected randomly then every 4th house on right side of the interviewed HHs was included until we covered the locality. According to available data, average number of HHs in one locality varies from 80-100. To over 382 HHs in each city we required nearly 15 HHs per area (=382/26). In each households, all eligible individuals were included for the study.

Supplementary Table S1. List of chronic conditions reported across the group.

		Slum (n=404) Percentage [95% CI]	Non-slum (n=695) Percentage [95% CI]	Total (n=1099) Percentage [95% CI]	P value#
Diseases	Hypertension	17.8[14.4-21.9]	15.0[12.5-17.8]	16.0[14.0-18.3]	0.213
	Arthritis	9.1[6.7-12.4]	4.1[2.8-5.8]	5.9[4.7-7.5]	0.001
	Acid peptic disease	5.4[3.6-8.1]	7.2[5.5-9.4]	6.6[5.2-8.2]	0.259
	Diabetes	11.1[8.4-14.6]	11.5[9.4-14.1]	11.4[9.6-13.4]	0.851
	Anemia	2.1[0.99-3.9]	0.7[0.3-1.7]	1.2[0.7-2.02]	0.062
	Infection	4.2[2.6-6.7]	1.6[0.9-2.8]	2.5[1.7-3.7]	0.008
	Asthma/COPD	11.9[9.1-15.4]	1.4[0.8-2.6]	5.3[4.1-6.7]	<0.001
	Weight problem	0	0.4 [0.1-1.33]	0.3[0.01-0.8]	
	Skin Disease	12.1[9.3-15.7]	3.2[2.1-4.8]	6.5[5.1-8.1]	<0.001
	Heart Disease	0.5 [0.1-1.9]	0.6[0.2-1.5]	0.5 [0.2-1.2]	0.861
	Kidney problem	0	1[0.5-2.1]	0.6[0.3-1.3]	0.043
	Mental Illness	0.2[0.03-1.7]	1.1[0.6-2.3]	0.8[0.4-1.6]	0.109
	Stroke	2.5[1.3-4.5]	2.3[1.4-3.7]	2.4[1.6-3.5]	0.856
Count	Zero	44.1[39.2-48.9]	72.2[68.8-75.4]	61.9[58.9-64.7]	<0.001
	One	43.6[38.8-48.4]	12.7[10.4-15.3]	24.0[21.6-26.6]	
	Two	4.7[3.0-7.3]	8.3[6.5-10.6]	7.0[5.6-8.7]	
	Three	6.7[4.6-9.6]	6.3[4.7-8.4]	6.5[5.1-8.1]	
	Four or more	1.0[0.3-2.6]	0.4[0.1-1.3]	0.6[0.3-1.3]	
Currently on medication	Yes	36.4 [31.8-41.2]	16.4 [13.8-19.3]	23.7 [21.3-26.3]	<0.001

#Chi square test, P value was significant at the level <0.05

Supplementary Table S2. List of heat illness across the group.

	Responses	Slum (n=404) Percentage [95% CI]	Non-slum (n=695) Percentage [95% CI]	Total (n=1099) Percentage [95% CI]	P value#
Ever experienced	Heat cramp	11.6 [8.8-15.1]	31.7 [28.3-35.2]	24.3 [21.8-26.9]	<0.001
	Heat exhaustion	48.8 [43.9-53.6]	46.3 [42.6-50.1]	47.2 [44.3-50.2]	0.436
	Heat Syncope	8.2 [5.9-11.3]	1 [0.5-2.1]	3.6 [2.7-4.9]	<0.001
	Heat rash	4.5 [2.8-6.9]	2.9 [1.9-4.4]	3.5 [2.5-4.7]	0.167
	Heat stroke	16.1 [12.8-20.1]	0.3 [0.07-1.15]	6.1 [4.8-7.7]	<0..001
Heat illness ever	Never	20.5 [16.9-24.8]	37 [33.4-40.6]	30.9 [28.3-33.7]	<0.001
	Once	70 [65.4-74.3]	43.9 [40.2-47.6]	53.5 [50.5-56.4]	
	Twice	9.2 [6.7-12.4]	19.1 [16.4-22.2]	15.5 [13.4-17.7]	
	Thrice	0.2 [0.03-1.74]	0	0.1 [0.01-0.6]	

#Chi square test, P value was significant at the level <0.05

Supplementary Table S3. Climatic data of study cities during the past four months (summer) of the study period.

	Bhubaneswar				Cuttack			
	March	April	May	June	March	April	May	June
Maximum temp (^{0}C)	38	41	42	43	39	41	42	43
Minimum temp (^{0}C)	21	20	24	24	21	22	20	24
Average temp (^{0}C)	28	30	32	32	28	30	30	30
Average Humidity (%)	73	75	70	80	73	74	70	80
